# Genomic and Virulence Investigations of a Novel Porcine Deltacoronavirus Strain Identified in South Korea

**DOI:** 10.1155/2023/5569675

**Published:** 2023-10-17

**Authors:** Duri Lee, Sangjune Shin, Guehwan Jang, Yunhee Gim, Hyun-Kyoung Son, Sang Chul Kang, Yongjoon Eo, Young-Gook Chae, Phil-Ok Koh, Hu-Jang Lee, Changhee Lee

**Affiliations:** ^1^College of Veterinary Medicine, Gyeongsang National University, Jinju 52828, Republic of Korea; ^2^ChoongAng Vaccine Laboratories, Daejeon 34055, Republic of Korea; ^3^Optipham Inc., Cheongju 28158, Republic of Korea; ^4^Nawoo Veterinary Group, Yangsan 50573, Republic of Korea; ^5^Kwangung Farm, Uiryeong 52110, Republic of Korea

## Abstract

Porcine deltacoronavirus (PDCoV) has emerged as a significant issue in multiple pork-producing countries. This study isolated a novel PDCoV strain, GNU-2105/KOR/2021, which caused a severe diarrhea outbreak with a high mortality rate among neonatal piglets in South Korea. The growth properties and sialic acid dependency of the GNU-2105 strain in cell culture were comparable to those of the 2016 domestic isolate, KNU-1607. Interestingly, phylogenetic analysis using the complete genome of GNU-2105 identified in 2021 demonstrated that this novel strain belongs to the US/South Korean/Japanese clade; however, it is more closely placed around the Chinese isolates. To investigate the potential pathogenic diversity between the previous and recent PDCoVs, we performed an experimental infection using conventional suckling piglets with KNU-1607 or GNU-2105. The KNU-1607-inoculated piglets suffered from acute, watery diarrhea; however, all piglets recovered and survived. In the KNU-1607-inoculated group, histopathological observation detected viral antigens in the jejunum and ileum. However, the virulence of the GNU-2105 virus was enhanced and presented severe clinical symptoms, including thin, transparent intestinal walls, with 100% mortality in piglets. Furthermore, viruses and severe villous atrophy were observed from the duodenum to the colon in all the piglets inoculated with GNU-2105 by quantitative RT-PCR and microscopic assessments, confirming the high enteropathogenicity of PDCoV in neonatal piglets. These findings could expand our understanding of the genetic and pathogenic variation of the PDCoV strain and highlight the necessity of vaccine development providing protection against virulent PDCoV.

## 1. Introduction

Porcine deltacoronavirus (PDCoV) is an emerging enteropathogenic swine coronavirus (CoV) that causes acute enteritis and mortality in newborn piglets [1, 2]. The clinical manifestations of PDCoV in neonates include anorexia, vomiting, watery diarrhea (mild-to-severe), dehydration, and growth retardation [3–5]. The disease outcomes are symptomatically comparable and clinically challenging to distinguish from those caused by other classical swine enteric CoVs, porcine epidemic diarrhea virus (PEDV) and transmissible gastroenteritis virus (TGEV) [6, 7]. However, PDCoV-infected pigs exhibit milder clinical signs and disease severity with lower mortality rates than those infected with PEDV and TGEV [4, 8, 9].

PDCoV belongs to the subgenus *Buldecovirus* of the genus *Deltacoronavirus* in the family *Coronaviridae* of the order *Nidovirales* [10]. The virus possesses a positive-sense, single-stranded RNA genome of approximately 25.4 kb in length, the smallest genome size among the porcine CoVs [2]. The PDCoV genome includes multiple open reading frames (ORFs). The ORF1a and ORF1b code for two polyproteins, 1a and lab, that is further processed by proteolytic cleavages to generate 15 nonstructural proteins (nsp2–16). By contrast, the downstream ORFs code for four conserved structural proteins, the glycosylated spike (S), envelope (E), membrane (M), and nucleocapsid (N) proteins, and three accessory proteins, namely nonstructural gene 6 (NS6), NS7, and NS7a [11–16].

PDCoV was discovered in 2012 by a territorial investigation study hunting CoVs that can infect mammalian and avian species and first emerged in pig farms in 2014 in the United States [2, 17]. Subsequently, PDCoV has been reported in several Asian pig-raising countries, including South Korea, Japan, mainland China, Thailand, Vietnam, and Laos. This indicated a global distribution with significant financial damages to the pork-producing nations [15, 18–21]. PDCoV was first announced in South Korea in 2014, shortly after its emergence in the US, and was genetically close to the US strains [22]. Our previous reports revealed a relatively high prevalence of PDCoV with a single infection and coinfection of PDCoV with PEDV across the nation, attracting particular attention to its potential ability to devastate pig populations in South Korea [23]. However, since the end of 2010, PDCoV outbreaks have occurred sporadically, causing unnoticed clinical and financial damages in domestic pork production. Furthermore, our animal inoculation experiment demonstrated a low enteropathogenicity of the PDCoV South Korean (KOR) strain, KNU-1607, without causing severe illness and mortality in neonatal piglets, suggesting that the fitness of this avian-originating CoV might have acclimated to the mammalian natural host [9]. Recently, a severe outbreak of PDCoV was reported in a large-scale commercial pig farm presenting with fatal watery diarrhea and high mortality in neonates. The absence of other etiological agents and high virulence suggested the potential emergence of a novel high-virulent PDCoV strain. To test this hypothesis, we isolated a novel PDCoV strain, GNU-2105/KOR/2021, from an affected farm and investigated its genotypic and phenotypic characteristics in vitro and in vivo.

## 2. Materials and Methods

### 2.1. Cells, Virus, and Antibodies

Swine testicular (ST) cells were grown in alpha minimum essential medium (*α*-MEM; Invitrogen, Carlsbad, CA) supplemented with 5% fetal bovine serum (FBS, Invitrogen) and Penicillin–Streptomycin (100×; Invitrogen). Porcine small intestinal epithelial cell line (IPEC-J2, a kind gift from Prof. Kang at Dankook University, South Korea [24]) was cultured in RPMI 1640 medium (Invitrogen) supplemented with 10% FBS, Penicillin–Streptomycin, 10 mM HEPES (Invitrogen), 1 mM sodium pyruvate (Invitrogen), and nonessential amino acids (100×; Invitrogen). The PDCoV KOR strain KNU-1607 was cultivated in ST or IPEC-J2 cells in virus growth medium (*α*-MEM or RPMI 1640 supplemented with Penicillin–Streptomycin, 10 mM HEPES (Invitrogen), and 5 *μ*g/ml of trypsin (USB, Cleveland, OH)) without FBS as described previously [9, 25, 26]. A monoclonal antibody (MAb) targeting the PDCoV N protein was described previously [9].

### 2.2. Clinical Case and Sample Collection

From November 2020 to April 2021, PDCoV outbreaks with fatal watery diarrhea leading to over 80% mortality in suckling piglets with sows also recurrently presenting black tarry diarrhea and anorexia occurred in a 1,000-sow commercial farrow-to-finish farm located in Gyeongbuk Province, South Korea. Small intestinal (SI, *n* = 4) and stool (*n* = 5) specimens collected from diarrheic piglets were submitted to our laboratory for diagnosis. Intestinal homogenates and fecal suspensions were prepared as described previously [27–29] and further subjected to RT-PCR for viral enteric pathogens [23, 30, 31]. PDCoV-positive specimens were filtered through a 0.22-*μ*m pore syringe filter (Millipore, Billerica, MA) and stored at −80°C until virus isolation and nucleotide sequencing were conducted.

### 2.3. Virus Isolation

PDCoV was isolated using ST cells in the presence of trypsin as described previously [9]. The viral supernatants harvested from the infected cells showing cytopathic effects (CPE) were then subjected to plaque purification. A single, well-separated plaque was collected, resuspended in virus growth medium, and labeled as a “passage 1 (P1)” PDCoV (GNU-2105-P1) stock. Subsequently, GNU-2105-P1 was sequentially propagated in ST cells for 4 passages (P5), and each passage GNU-2105 strain was aliquoted and stored at −80°C until usage. The P5 stock of each PDCoV strain was used for subsequent experiments unless otherwise indicated.

### 2.4. Immunofluorescence Assay

ST or IPEC-J2 cells were mock-infected or infected with KNU-1607 (P5) or GNU-2105 (P5) in the presence of trypsin at a multiplicity of infection (MOI) of 0.1 for 1 hr. At 6 hr postinfection (hpi), infected cells were subjected to immunofluorescence assay (IFA) using the PDCoV *N*-specific MAb as described previously [25, 26]. The stained cells were visualized under a Leica DM IL LED fluorescence microscope (Leica, Wetzlar, Germany).

### 2.5. Plaque Assay

ST or IPEC-J2 cells grown in six-well tissue culture plates were inoculated with each virus suspension containing trypsin (MOI of 0.1) at 37°C for 1 hr. After adsorption, the infected cells were overlaid with 2 ml of virus growth medium premixed with 1.5% Bacto Agar (Difco, Detroit, MI) and propagated at 37°C for 1 day until adequately sized viral plaques were developed. After the removal of the agar medium at 24 hpi, the plaques were fixed with 7% paraformaldehyde and stained with 1% crystal violet in 5% ethanol.

### 2.6. Quantitative Real-Time RT-PCR

Viral RNA was extracted from virus supernatants from infected ST or IPEC-J2 cells using *i*-TGE/PED Detection Kits (iNtRON Biotechnology, Seongnam, South Korea). PDCoV M gene-based real-time RT-PCR (rRT-PCR) was conducted using One Step TB Green PrimeScript RT-PCR Kits (TaKaRa, Otsu, Japan) as described previously [9, 25, 26]. The reaction mixtures were amplified using a CronoSTAR 96 Real-Time System (Clontech, Mountain View, CA), and the results were analyzed using the software as described previously [32].

### 2.7. Virus Titration

ST or IPEC-J2 cells were inoculated with each PDCoV strain (P5) as described above. The culture supernatant was harvested at 3, 6, 9, 12, and 24 hpi. Virus titers were measured by limiting the dilution in duplicate cells as described previously [9, 25, 26], and then 50% tissue culture infectious dose (TCID_50_) per ml was determined using the Reed–Muench method [33].

### 2.8. Neuraminidase Treatment Infectivity Assay

Neuraminidase (NA) treatment infectivity assay was performed as described previously with some modifications [34–36]. In brief, ST or IPEC-J2 cells grown in 96-well tissue culture plates for 24 hr were pretreated with 50 *μ*l of 200 mU NA from *Clostridium perfringens* (Roche Diagnostics, Mannheim, Germany) at 37°C for 2 hr. The cells were then inoculated with each virus strain (P5) containing trypsin at an MOI of 1 for 1 hr and further cultured in *α*-MEM or RPMI 1640 supplemented with 5% FBS for 5 hr. The number of PDCoV-infected cell foci was measured by IFA, and the fluorescence intensity of IFA was determined using a microplate fluorometer (Fluoroskan FL; Thermo Scientific, Waltham, MA).

### 2.9. Genetic and Phylogenetic Analysis

The full-length genomic nucleotide sequences of the GNU-2105-SI, -P1, and -P5 strains were determined using Sanger sequencing, as previously described [9, 15, 23]. Nine cDNA fragments covering the complete viral genome were amplified and sequenced as described previously [23]. The 5′ and 3′ ends of the genome of each isolate were determined by rapid amplification of cDNA ends (RACE), as described previously [37]. The whole-genome sequence data of the GNU-2105-SI, -P1, and -P5 viruses were deposited at NCBI GenBank with accession numbers OQ566226-8. A total of the 123 genomic sequences of global PDCoV isolates were used in sequence alignments and phylogenetic analyses as described elsewhere [38–40].

### 2.10. Animal Challenge Experiments and Clinical Examinations

The animal study protocol was approved by the Institutional Animal Care and Use Committee (IACUC) of Gyeongsang National University (IACUC no. GNU-221212-P0176) and performed at the GNU Animal Facility as described previously [27, 29, 35, 41, 42]. Fifteen neonates (3-day-old at the beginning of the study) were acquired from commercial crossbred sows (Great Yorkshire × Dutch Landrace) at a conventional breeding herd with no previous record of PDCoV exposure or vaccination. All animals tested negative for known porcine enteric viruses by virus-specific rRT-PCRs on rectal swabs [23, 30, 31]. Animals were randomly allocated by weight to three experimental groups (KNU-1607, GNU-2105, and mock inoculation) and housed in three separate rooms. The piglets were fed commercial milk replacer (three to four times daily) and had ad libitum access to water throughout the study [27, 29, 35, 41, 42]. After a 2-day acclimation period, the 5-day-old animals were challenged orally with KNU-1607 (*n* = 5) or GNU-2105 (*n* = 6) at a dose of 10^4.0^ TCID_50_/piglet (equivalent to 100 median diarrhea doses of KNU-1607 in neonates) [9]. The sham group (*n* = 4) were administrated with cell culture medium as a placebo. Clinical signs, including vomiting, diarrhea, and death, were recorded daily for 7 days postinoculation (DPI). Rectal swab samples were collected before inoculation and thereon daily and subjected to rRT-PCR to quantify viral shedding in feces. The virus titers (genomic copies/ml) in the fecal samples were calculated for all pigs in each group as described previously [9]. A clinical significance score (CSS) was determined based on stool consistency for 7 DPI as described previously [29, 35, 41, 42]. The dead animals were necropsied at the time of death, while the surviving pigs from each group were euthanized at 7 DPI for postmortem examinations.

### 2.11. Histopathology and Direct Immunofluorescence

Intestinal tissue (duodenum, proximal jejunum, ileum, cecum, and colon) samples (<3 mm thick) were collected from each piglet, fixed in 10% formalin for 24 hr at RT, and embedded in paraffin according to standard laboratory protocols as described previously [27, 29, 41, 42]. The deparaffinized intestinal tissue sections were stained with hematoxylin and eosin (H&E; Sigma–Aldrich) for histopathology or subjected to direct immunofluorescence (DIF) for viral antigen detection as described elsewhere [27, 29, 35, 41, 42]. The severity of villus atrophy was determined by quantifying the ratio between villous height and crypt depth (VH : CD) throughout the H&E-stained tissue sections, and the mean ratio of VH : CD in each small intestine segment was calculated as described previously [43].

### 2.12. Statistical Analysis

All values are presented as the mean ± standard deviation of the mean difference (SDM). Statistical analyses were performed using the GraphPad Prism 7 software package (GraphPad Software, San Diego, CA). If the *P*-value is 0.05 or lower, the result is deemed to be statistically significant.

## 3. Results

### 3.1. Virus Isolation and In Vitro Phenotypic Characteristics of PDCoV Strain GNU-2105

**Figure 1:**
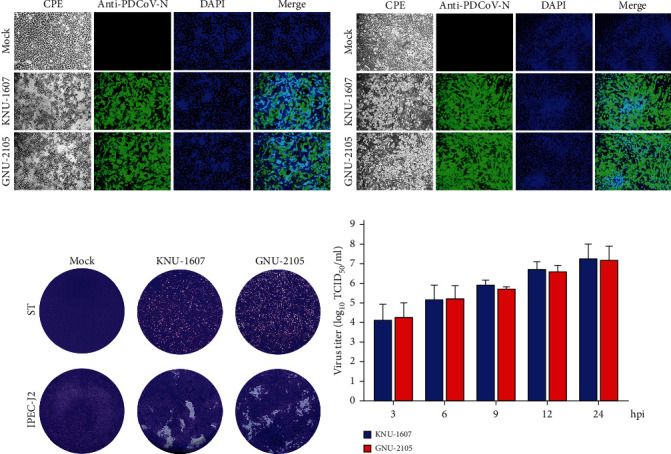
Cytopathology and growth properties of PDCoV strains KNU-1607 and GNU-2105. (a and b) Phenotypic characteristics of KNU-1607 and GNU-2105. ST (a) or IPEC-J2 (b) cells were mock-infected or infected with each virus at an MOI of 0.1. PDCoV-specific CPE was monitored daily, and cells were photographed at 6 hpi using an inverted microscope at a magnification of 200× (left panels). For immunostaining, infected cells were fixed at 6 hpi and incubated with MAb against the PDCoV N protein, followed by incubation with Alexa green-conjugated goat antimouse secondary antibody (second panels). The cells were then counterstained with DAPI (third panels) and examined under a fluorescence microscope at 200× magnification. (c) Representative images showing the plaque morphology phenotypes of KNU-1607 and GNU-2105. Monolayers of ST (top panels) or IPEC-J2 (bottom panels) cells were infected with each strain. The cells were maintained in agar overlay medium and incubated for 1 day. Plaques were stained with crystal violet and photographed at 24 hpi. (d) Growth kinetics of KNU-1607 and GNU-2105. At the indicated time points postinfection, culture supernatants were harvested, and virus titers were determined. The results are expressed as the mean of three independent experiments performed in duplicate, and the error bars show the SDM.

The initial diagnostic tests confirmed that all clinical SI and fecal samples were PDCoV positive. By contrast, no other viral enteric pathogens were detected, including PEDV, TGEV, and porcine rotavirus (PRV) (data not shown). We then attempted to inoculate ST cells with PDCoV-positive clinical samples for virus isolation, and the viral-induced CPE was observed daily. The PDCoV strain designated GNU-2105 was isolated from the SI homogenate (GNU-2105-SI) prepared from a piglet naturally infected with the strain and cultivated in cell culture. Upon CPE observation, the culture supernatants were cloned by plaque purification, and the purified virus (GNU-2105-P1) was passaged up to four passages to enhance the virus titers. In infected ST cells from passage 1, the GNU-2105 virus produced distinct CPE typical of a PDCoV infection, such as rounding, clumping together in clusters, and cell detachment, as described previously [9]. In later passages of GNU-2105, CPE was visible at 6 hpi and became pronounced by 12 hpi, comparable to that in KNU-1607-P5. Virus propagation was verified by detecting PDCoV antigens with IFA using the PDCoV *N*-specific MAb. The apparent staining was localized to the cytoplasm of infected cells. By contrast, neither CPE nor *N*-specific staining was manifest in mock-inoculated ST cells. These growth properties of both strains were reproduced in IPEC-J2 cells. Examples of CPE and resultant IFA images from ST and IPEC-J2 cells infected with KNU-1607-P5 or GNU-2105-P5 are shown in Figures 1(a) and 1(b). As seen in Figure 1(c), the plaques of KNU-1607 and GNU-2105 were comparable in size and morphology in both cell lines. The amount of viral genome in each strain was evaluated and compared. The mean Ct values were comparable between KNU-1607 and GNU-2105, showing 13.57 and 12.65, respectively. The viral replication kinetics study in ST cells indicated that both viruses displayed indistinguishable growth curves and comparably achieved maximum titers >10^6^ TCID_50_/ml at 12 hpi (Figure 1(d)). In addition, both strains replicated efficiently in IPEC-J2, displaying similar growth kinetics to those in ST cells (data not shown).

**Figure 2:**
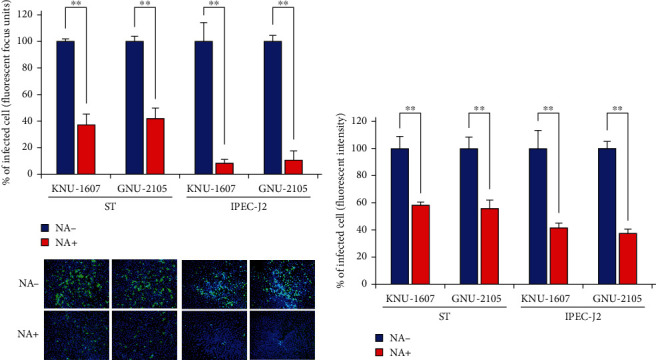
Dependency of PDCoV strains on cell surface acids for infection. (a) Neuraminidase treatment infectivity assay. ST or IPEC-J2 cells were pretreated with neuraminidase (NA+) or PBS (NA−) at 37°C for 2 hr and then inoculated with KNU-1607 or GNU-2105 at an MOI of 1 for 1 hr in the presence of trypsin. Cells were maintained in *α*-MEM or RPMI containing 5% FBS and subjected to IFA at 5 hpi as described in the legend of Figure 1(a). The percentage of fluorescent foci of sialic acid-dependent infection was calculated by counting the infected cells in 200× microscopic fields. IFA images acquired with a fluorescence microscope are presented at the bottom. (b) Fluorescence intensity measurement. The fluorescence emission of the IFA staining, as described above, in the wells was detected by fluorometry, and the percentage of fluorescent intensity was plotted. The values shown are the mean of three independent experiments, and the error bars denote the SDM.  ^*∗*^ ^*∗*^*P* < 0.001.

Sialic acid (SA) on the cell surface serves as a receptor for PDCoV and augments infection efficiency [44]. Therefore, we evaluated the role of SA in the cells infected with each PDCoV strain. To accomplish this, we performed NA treatment of ST or IPEC-J2 cells that can deplete SA from the cell surface before viral infection. The NA-treated cells were infected with each PDCoV-P5 stock containing trypsin, cultivated in the medium formulated with FBS to prevent the action of trypsin (which could restrict viral dissemination), and subjected to IFA at 5 hpi. In comparison to the NA-untreated and KNU-1607-infected control cells, the quantity of fluorescent foci was significantly diminished (62.8% and 91.7% decreases) in the NA-treated and infected ST and IPEC-J2 cells, respectively (Figure 2(a)). Similarly, NA pretreatment significantly affected the infectivity of GNU-2105. We observed 58.2% and 89.6% reductions in ST and IPEC-J2 cells pretreated with NA, respectively, compared to the respective NA-untreated and infected control cells. The level of the NA treatment-dependent infectivity of each virus was also corroborated by fluorometry (Figure 2(b)). These data demonstrated the importance of both PDCoV strains on SA present on the cell surface for infection.

### 3.2. Genetic and Phylogenetic Characterization of PDCoV Strain GNU-2105

We determined and analyzed the full-length genomic sequences of the GNU-2105-SI isolate and the GNU-2105-P1 and -P5 viruses grown in cell culture. The entire genomes of the GNU-2105-SI, -P1, and -P5 strains consisted of 25,422 nucleotides (excluding the 3′ poly(A) tail) long and displayed organization identical to all previously sequenced PDCoVs. The entire genome sequences of the GNU-2105-P1 and -P5 viruses were compared to the original GNU-2105-SI, and the results are summarized in Table 1. Compared to GNU-2105-SI, GNU-2105-P1, and -P5 showed identical six nucleotides (nt) differences at positions 3,444, 9,567, 17,015, 21,115, 21,209, and 21,247. Among these, five were nonsilent mutations, causing one, one, and three amino acid (aa) substitutions in the ORF1a, ORF1b, and S coding regions, respectively. The three aa mutations at positions 598 (Ser to Pro), 629 (Phe to Ser), and 642 (Gln to Lys) in the S coding region were conserved through passage 10 (GNU-2105-P10). Cell culture-adapting mutations were absent in the regions coding for E, M, N, and accessory genes.

The complete genome sequence analysis showed that the GNU-2105 strain has 99.4% homology with a PDCoV KOR prototype strain (KNU14-04) at the genome level, resulting from 1-nt, 35-aa, and 8-nt variations in 5′ UTR, protein-coding ORFs, and 3′ UTR (Table 2). Aligning the genome sequences indicated that GNU-2105 is most closely related to the US, KOR, and Japanese (UKJ) isolates, sharing nt identities of 99.0%–99.5% with 131–248 nt differences at the genome level (Table 3). By contrast, the GNU-2105 strain was distantly related to isolates reported in China and Southeast Asian (SEA) countries, including Vietnam, Thailand, and Laos. Moreover, GNU-2105 showed the lowest identity to SEA strains, ranging from 96.9% to 97.9% with 546–782 nt variations at the genome level (Table 3). The UKJ strains possessed two insertion (IN) signatures in the S gene (at nt positions 19,476–19,478 or aa position 52 in S) and the 3′ UTR (nt positions 25,048–25,050) when compared to the PDCoV prototype strain HKU15-155 [15]. For GNU-2105, the former IN signature in the S gene was identical, while the latter “TTA” IN signature in the 3′ UTR was modified to TCT. In addition, the GNU-2105 strains possessed multiple point mutations in the 3′ UTR, including C25086T and G25096A, which are common in the Chinese strains.

**Figure 3:**
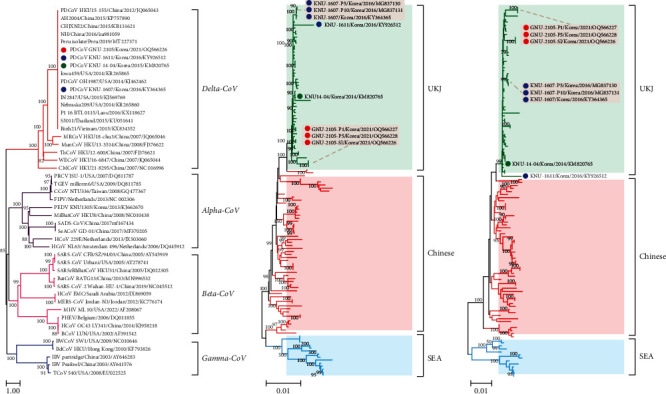
Phylogenetic analysis based on the full-length genome sequences of four coronavirus (CoV) genera (*Alpha-CoV*, *Beta-CoV*, *Gamma-CoV*, and *Delta-CoV*) (a) and the nucleotide sequences of the complete genomes (b) and the S genes (c) of PDCoV strains. Multiple sequence alignments were performed using ClustalX software, and phylogenetic trees were constructed from the aligned nucleotide sequences using the neighbor-joining method. Numbers at each branch are bootstrap values greater than 50% based on 1,000 replicates. The names of the strains, countries along with the dates (year) of isolation, GenBank accession numbers, and clades proposed in this study are shown. Individual clades are shaded in different colors: yellow-green (UKJ), orange (Chinese), and sky-blue (SEA). South Korean (KOR) PDCoV strains are also shaded in gray. Blue circles indicate the KNU-1607 strains; red circles indicate the GNU-2105 strains identified in this study; green circles indicate the KOR prototype strain KNU14-04. Scale bars indicate nucleotide substitutions per site.

We then performed phylogenetic analyses using the nt sequences of the complete genome and the S gene of the GNU-2105 strain and those accessible from the GenBank database, with representative CoV sequences from other genera (Figure 3). The phylogeny based on the full-length genome sequence confirmed that the GNU-2105 strains were grouped into a swine-origin clade in the *Deltacoronavirus* genus, which was diverse from avian-origin deltacoronaviruses (Figure 3(a)). Further phylogenetic tree based on the entire genomes or the S genes of PDCoV exhibited three geographically disjunct clades: UKJ, Chinese, and SEA clades (Figures 3(b) and 3(c)). Although all previous and recent KOR strains belonged to the UKJ clade, they were situated on different branches, suggesting ongoing viral evolution. Interestingly, the complete genome-based phylogeny showed that the GNU-2105 strains were more closely placed (Figure 3(b)), whereas the S gene-based phylogeny indicated that they were more distantly placed from the Chinese clade (Figure 3(c)). Taken together, the phylogenetic results suggested that the contemporary strains identified in 2021 are dissimilar to the past emergent viruses in South Korea.

### 3.3. Pathogenicity of PDCoV Strain GNU-2105 in Neonatal Piglets

Since the GNU-2105 strain was identified from a conventional farrow-to-finish swine herd that suffered from severe-scale neonatal death, we were interested in evaluating its biological characteristics in the natural host. Thus, the pathogenicity of the 2016 (KNU-1607) and 2021 (GNU-2105) isolates were investigated and assessed after experimental challenge of neonates. Fifteen piglets were assigned into three groups: the first one (five animals) challenged with KNU-1607 (group 1), the second one (six animals) challenged with GNU-2105 (group 2), and the last one (four animals) inoculated with the cell culture medium (sham). We recorded the clinical symptoms thrice daily and collected fecal samples before inoculation and daily after inoculation for the entire experimental period. During acclimatization, all animals were vigorous and developed no clinical signs with normal stool consistency, and their rectal swabs tested negative for PDCoV RNA.

**Figure 4:**
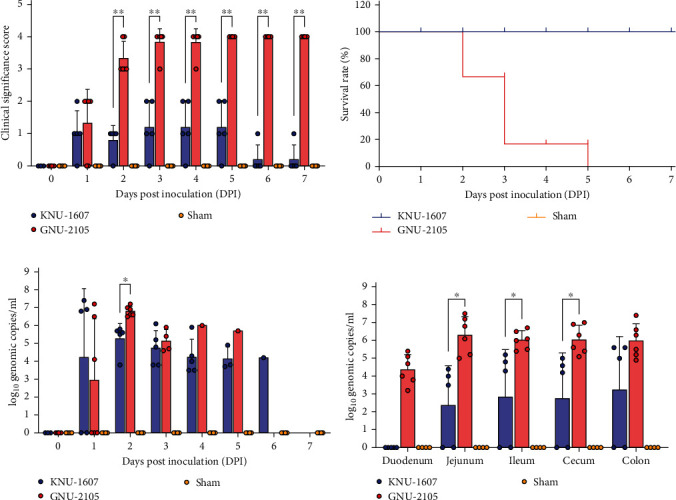
Virulence of PDCoV strains in neonatal piglets. (a) Clinical significance scores (CSS) of each group. The CSS of individual pigs from each group was measured using the scoring criteria for diarrheal severity: 0, normal and no diarrhea (mean Ct values > 35); 1, mild and fluidic feces; 2, moderate mucus to watery diarrhea; 3, severe watery and projectile diarrhea (mean Ct values < 20); 4, death. (b) Survival curves. Individual piglets were monitored, and the survival rates of each group until 7 DPI were plotted. (c) Fecal PDCoV shedding profile of each group. PDCoV RNA titers (log_10_ genomic copies/ml) in rectal swaps at the indicated sampling time points were determined using rRT-PCR. (d) PDCoV distribution in the intestinal tissues. PDCoV RNA loads (log_10_ genomic copies/ml) in each intestinal tissue collected at necropsy (performed upon death or after euthanasia at 7 DPI) were determined using rRT-PCR. The mean values of each group at each time point are presented, and error bars denote the SDM. *P* values were calculated by comparing the data between the KNU-1607 virus- and GNU-2105 virus-inoculated groups.  ^*∗*^*P*=0.001 − 0.05;  ^*∗*^ ^*∗*^*P* < 0.001.

In the sham-inoculated group, none of the animals presented clinical manifestations of PDCoV throughout the challenge experiment. However, most KNU-1607-challenged piglets (4/5) in group 1 exhibited diarrheic feces at 1 DPI (mean CSS = 1.0) and continued to experience mild to moderate diarrhea by 5 DPI (mean CSS = 0.8–1.2; Figure 4(a)). Furthermore, nearly all animals (4/5) in group 1 recovered at 6 DPI, and all survived throughout the observation period (Figure 4(b)). Similarly, except for one pig in group 2, most animals (5/6) inoculated with GNU-2105 displayed clinical disorders (e.g., lethargy, anorexia, and moderate diarrhea) by 1 DPI (mean CSS = 1.3). Strikingly, all piglets in group 2 subsequently underwent severe watery diarrhea (mean CSS > 3.0; Figure 4(a)), and PDCoV-associated mortality appeared in all challenged piglets in group 2 by 5 DPI: 2/6, 3/6, and 1/6 at 2, 3, and 5 DPI, respectively (Figure 4(b)).

In group 1, 60% of piglets (3/5) inoculated with KNU-1607 tested positive for PDCoV genome, as verified by rRT-PCR in stool specimens, by 1 DPI with a mean titer of 10^7.1^ genomic copies/ml. All pigs in group 1 shed viruses in their feces by 2 DPI with a mean titer of 10^5.3^ genomic copies/ml. Afterward, viral shedding steadily decreased, ranging from 10^0.8^ to 10^4.7^ genomic copies/ml until 6 DPI and reached a level undetectable by rRT-PCR at 7 DPI (Figure 4(c)). Similarly, three GNU-2105-challenged piglets (3/6) in group 2 shed PDCoV in their feces with a mean titer of 10^6.0^ genomic copies/ml at 1 DPI. However, all animals in group 2 shed rising viral load in their feces with a mean titer of 10^6.8^ genomic copies/ml at 2 DPI and showed high fecal PDCoV RNA titers ranging from 10^5.2^ to 10^6.0^ genomic copies/ml until death (Figure 4(c)). The animals in the sham group stayed healthy without diarrheic feces and detectable fecal viral shedding throughout the study. PDCoV RNA load was also determined in the intestinal tissues collected during necropsy. Three piglets inoculated with KNU-1607 in group 1 tested positive for PDCoV in most intestinal segments, except the duodenum, and the remaining animals (2/5) were confirmed as negative for PDCoV in all intestinal tissues. By contrast, PDCoV RNA was present in all five intestinal segments of the GNU-2105-challenged piglets in group 2. Overall, each intestinal tissue of the piglets challenged with GNU-2105 in group 2 contained significantly higher titers of PDCoV, with mean titers ranging from 10^4.4^ to 10^6.3^ genomic copies/ml, compared with those of the piglets in group 1 (Figure 4(d)).

**Figure 5:**
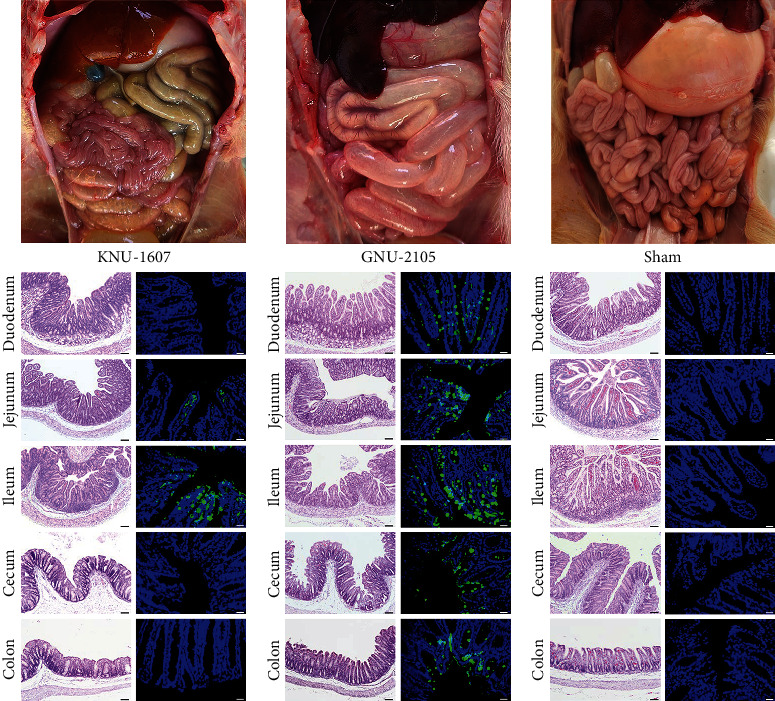
Macroscopic and microscopic intestine lesions in piglets inoculated with KNU-1607 (a) or GNU-2105 (b) and in sham control animals (c). Small and large intestines of individual piglets from each group were examined for gross lesions. Representative necropsy images are presented at the top of each panel. Note that piglets inoculated with GNU-2105 typically presented with thin, transparent intestinal walls. Hematoxylin and eosin-stained intestinal tissue sections from representative piglets in each group are shown at the bottom left of each panel (100× magnification, scale bar = 100 *µ*m). Severe villous atrophy can be observed in small intestinal sections of GNU-2105-inoculated piglets. DIF analysis results showing PDCoV antigen in each intestinal tissue section from representative piglets in each group are presented at the bottom right of each panel (200× magnification, scale bar = 50 *µ*m). Immunofluorescence staining of PDCoV N proteins was detected in the epithelial cells of the intestinal sections in piglets inoculated with KNU-1607 and GNU-2105. No PDCoV antigen was identified in the intestine of the mock-inoculated piglets.

All piglets in group 2 that died at 2–4 DPI were necropsied upon death. By contrast, the remaining animals that survived in groups 1 and 3 were euthanized after termination of the trial for postmortem assessment. The animals in group 1 that survived against KNU-1607 challenge macroscopically displayed mild intestinal lesions at necropsy (Figure 5(a)). Furthermore, histopathological and DIF examinations revealed the presence of villous atrophy and viral antigens only in the jejunum and ileum tissues of piglets infected with KNU-1607. In group 2, the GNU-2105-inoculated piglets showed severe pathological lesions in their intestines upon necropsy after death. Due to villous atrophy, the intestines were distended and presented with thin and transparent intestinal walls (Figure 5(b)). The histopathological evaluation indicated that the small intestine of all dead piglets in group 1 was characterized by acute viral enteritis with villous contraction and vacuolation. DIF test detected PDCoV N proteins in the atrophied villous epithelium in the small (duodenum to ileum) and large (cecum and colon) intestines from the GNU-2105-inoculated group. In contrast, neither macroscopic nor microscopic intestinal changes nor immunofluorescence-stained cells were detected in the sham-inoculated negative control group (Figure 5(c)).

**Figure 6:**
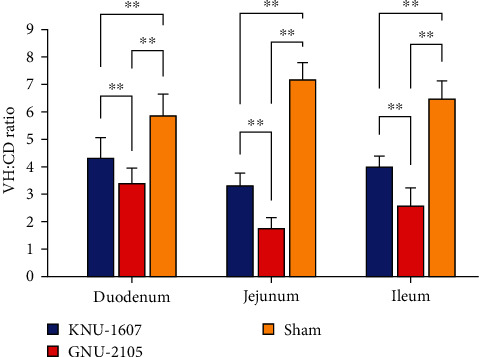
VH : CD ratios of piglets inoculated with KNU-1607 or GNU-2105. Ten villi and crypts of each small intestinal section were measured. The mean VH : CD ratios of individual small intestine section in each group are presented, and error bars denote the SDM.  ^*∗∗*^*P* < 0.001.

Moreover, the mean (± SDM) VH : CD ratios of the duodenum, jejunum, and ileum varied statistically among the groups (Figure 6); the GNU-2105 challenge group had the lowest VH : CD ratio (1.78 ± 0.34–3.44 ± 0.49), the KNU-1607 challenge group had the midrange ratio (3.34 ± 0.41–4.34 ± 0.69), and the sham control group had the highest ratio (5.90 ± 0.71–7.21 ± 0.55). These observations corroborated the increased virulence of GNU-2105 compared to KNU-1607. Collectively, our data demonstrated that the contemporary PDCoV strain GNU-2105 was highly enteropathogenic in neonatal piglets.

## 4. Discussion

PDCoV is a novel swine enteric deltacoronavirus that has threatened the pork industry development since the last decade [2]. Although birds serve as primary reservoirs for deltacoronaviruses, the avian-origin CoVs can cross the species barrier to infect and adjust to some mammals, including pigs. Enteropathogenicity of this emerging virus in both conventional and gnotobiotic animals was elucidated under various experimental situations; however, PDCoV-infected pigs exhibited milder clinical outcomes (i.e., disease severity, morbidity, and mortality) than those infected with other enterotropic swine CoVs, PEDV and TEGV [1, 3, 4, 8, 9, 45]. Although a single infection of PDCoV in naturally infected piglets is commonly associated with diarrhea, coinfections of PDCoV with other enteric viruses, such as PEDV or/and PRV, are frequent with natural infections, resulting in high neonatal mortality and substantial economic losses for the pig herd [6, 46]. Furthermore, PDCoV can infect other animal species, including calves, chickens, turkey poults, and mice, under experimental conditions [47–50], and single infections of PDCoV among Haitian children have also been reported [51], creating a potential risk of cross-species transmission. In South Korea, the first emergence of PDCoV was reported in 2014 [15]. Despite the high prevalence of its single and coinfections with PEDV in diarrheal samples, only small-scale PDCoV occurrences have been reported intermittently across mainland South Korea and resulted in death with low mortality following the outbreaks [23]. Moreover, a previous experimental infection study confirmed that PDCoV is low-pathogenic in conventional suckling piglets with no mortality, indicating onward evolutionary adaptation for PDCoV transmission and infection in pig populations following avian-to-mammalian spillover [9]. Recently, a PDCoV-related diarrhea outbreak emerged in the southeastern region of South Korea and caused a mass incidence of neonatal mortality (more than 80%), suggesting a pathogenic shift in PDCoV. We characterized the full-length genome of PDCoV responsible for this severe outbreak and evaluated its pathogenicity in neonatal piglets.

In the current study, a novel PDCoV strain, GNU-2105, was first isolated from the small intestine of a piglet that died after a PDCoV infection, and its phenotypic and genotypic traits were investigated. The GNU-2105 strain was cytopathogenic in ST cells from passage 1 and at passage 5, displayed more severe and rapid CPE characterized by rounding, clumping, and detaching of infected cells. The initial infectious titers of GNU-2105 ranged from approximately 5–6 log_10_ TCID_50_/ml and mounted after several passages, reaching over 7 log_10_ TCID_50_/ml. These growth properties, including cytopathology, viral titers, and growth kinetics, were replicated in IPEC-J2 cells. Yuan et al. [44] revealed that PDCoV could bind to SA to elevate its infection efficiency, although SA is dispensable during PDCoV infection. This study confirmed that KNU-1607 and GNU-2105 strains were dependent on SA for infection of ST and IPEC-J2 cells, and the degree of the SA-dependent infection was similar between the two strains; however, it is noteworthy that the removal of SA affected the infectivity of PDCoV in IPEC-J2 cells more than that in ST cells. Collectively, the phenotypic features were comparable for past KNU-1607 and contemporary GNU-2105 strains in two susceptible porcine ST and IPEC-J2 cell lines.

The whole genome sequences of three GNU-2105 strains (GNU-2105-SI, -P1, and -P5) were determined by the Sanger and RACE methods. Compared to the original SI isolate (GNU-2105-SI), six nt variations in protein-coding regions were identified for the first passage (GNU-2105-P1) in cell culture, which resulted in five nonsynonymous mutations in nsp6 (1), nsp14 (1), and S (3). The five aa changes were maintained at the corresponding positions of GNU-2105-P5, suggesting their potential significance for adapting the field virus to in vitro growth. In addition, the three mutations (S598P, F629S, and Q642K) in the S protein were sustained for 10 passages in the cell culture. Moreover, sequence comparisons with other PDCoV strains revealed that GNU-2105 isolates were most closely related to the UKJ lineage strains with 99.0%–99.5% identities but were most distinctly related to the SEA lineage strains with 96.9%–97.9% identities at the genome level. Furthermore, like the UKJ strains, GNU-2105 contained two IN signatures, comprising “AAT (Asp)” IN in the S gene and “TCT” in the 3′ UTR, compared to the prototype HKU15-155 strain [15]. Interestingly, the 3′ UTR of GNU-2105 was mutated to be more closely related to that in the Chinese strains. Subsequent phylogenetic assessments based on the full-length genome and the complete S gene formed a similar tree structure that created three geographically disjunct UKJ, Chinese, and SEA clades, revealing that the GNU-2105 strains are clustered together with the UKJ clade. However, the GNU-2105 strains were more adjacently situated with the Chinese clade in the phylogenetic tree constructed based on the whole genome. Therefore, the genetic and phylogenetic analyses indicated that PDCoV undergoes continuous genetic drift under field circumstances.

PDCoV can cause several deaths (40%–80% mortality rate) among suckling piglets following outbreaks [52–54]. The GNU-2105 virus is an emerging PDCoV strain isolated from a commercial farrow-to-finish farm experiencing a PDCoV-associated diarrhea outbreak with higher neonatal mortality (>80%). To demonstrate whether or not the GNU-2105 strain could induce such fatal outcomes with high mortality, conventional neonatal piglets (5-day-old) were orally inoculated with the virus. Our previous study demonstrated that experimental oral inoculation of piglets with the KNU-1607 strain caused mild clinical signs and no mortality throughout the experiment, indicating that the virus was low enteropathogenic in neonates [9]. In this study, the low pathogenic features of KNU-1607 were reproduced under the same experimental conditions. The KNU-1607-associated diarrheal disease was mild, with spontaneous recovery in neonatal piglets that can shed viral genetic material in their feces from 1 to 6 DPI. This short-term fecal shedding of PDCoV in clinically affected pigs could be correlated with low morbidity, which can mirror the status of sporadic PDCoV occurrence in South Korea. Consistent with this result, no PDCoV RNA was identified in entire intestinal segments (from the duodenum to the colon), in some KNU-1607-infected pigs at 7 DPI (upon necropsy). Gross lesions with virus infection were observed in the intestinal tissues of some KNU-1607-inoculated pigs at 7 DPI; however, histopathological lesions and PDCoV antigens were observed in the villous epithelium of jejunum and ileum. By contrast, all neonates challenged with GNU-2105 experienced fatal watery diarrhea from 2 DPI to died at 2–5 DPI. Although the level of fecal viral RNA shedding in GNU-2105-inoculated piglets during their survival was comparable to the KNU-1607-inoculated group, higher viral RNA copies were detected in all intestinal tissues in the entire GNU-2105-challenged group at necropsy. Furthermore, all animals necropsied at death in the GNU-2105-infected group showed severe lesions and DIF antigens in the small and large intestines according to macroscopic and microscopic observations, respectively. Our findings indicated the similarities in severity and fatality of GNU-2105 in conventional neonates in the field and experimental circumstances, demonstrating that the contemporary strain, GNU-2105/KOR/2021, is highly virulent in newborn pigs, entirely distinctive of the virulence of the past KNU-1607 virus.

**Figure 7:**
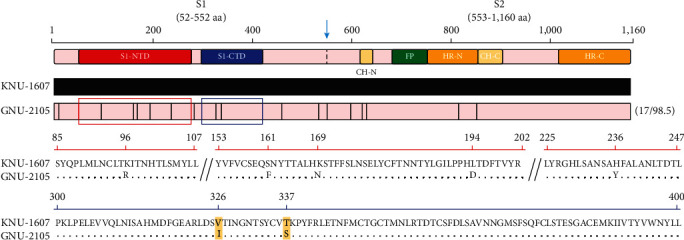
Schematic diagram of genetic variations in the S gene between KNU-1607 and GNU-2105. The top illustration represents the organization of the S protein, featuring S1 and S2 subunits that contain an N-terminal domain (S1-NTD), a C-terminal domain (S1-CTD), an N-terminal central helix (CH-N), a C-terminal central helix (CH-C), a fusion peptide (FP), an N-terminal heptad repeat (HR-N), and a C-terminal heptad repeat (HR-C). Red- and blue-highlighted areas in the diagram depict sialic acid-binding (S1-NTD, residues 52–277) and receptor binding (S1-CTD, residues 300–419) domains of PDCoV. Lightly shaded areas (GNU-2105) are identical to KNU-1607 (thick horizontal black line), and the vertical black bars represent one amino acid sequence that is divergent from that of KNU-1607. Red and blue boxes indicate the S1-NTD and S1-CTD regions, respectively. The digits in parentheses on the right indicate the number of amino acid changes and the percent identity compared with KNU-1607. The amino acid sequence alignments of the partial S1-NTD (red line, residues 85–247) and S1-CTD (blue line, residues 300–400) between KNU-1607 and GNU-2105 are presented at the bottom. Two amino acid changes (V326I and T337S) in the S1-CTD are highlighted in yellow.

Considering the CoV S protein as the major viral determinant for cell entry, host range, and tissue tropism, S glycoprotein mutations can drive alterations in the phenotypic traits (i.e., receptor usage and virulence) of PDCoV. The S protein of PDCoV can be separated into S1 and S2 subunits that function in receptor binding and virus-cell membrane fusion, respectively [55]. The PDCoV S1 subunit contains two main functional regions, an N-terminal domain (S1-NTD) and a C-terminal domain (S1-CTD): the former exhibits SA-binding activity to promote cell entry, while the latter functions as the receptor binding domain (RBD) bound to porcine aminopeptidase N (pAPN) [56–59] (Figure 7). As depicted in Figure 7, GNU-2105 possessed five aa variations (K96R, S161F, K169N, H194D, and H236Y) in the S1-NTD compared to KNU-1607; however, both strains had the equivalent ability to use SA as an attachment receptor, suggesting the irrelevance of those genetic changes to the PDCoV SA-binding employment. Furthermore, the depletion of SA by NA treatment reduced but could not completely block PDCoV infectivity, indicating that SA is dispensable during PDCoV infection. This phenomenon might result from the dependence of PDCoV on a major pAPN receptor to guarantee the productive survival and spread of the virus [59]. Compared to KNU-1607, two aa mutations (V326I and T337S) emerged in the S1-CTD of GNU-2105 (Figure 7), which may strengthen the interaction between PDCoV and pAPN. Interestingly, the dyad changes cannot be ascertained in the genome sequences of the global PDCoV strains deposited in the GenBank database. Although more detailed studies are required, we assume that the dyad drift in the S1-CTD enhances virus affinity to pAPN, which in turn can offer some benefits (e.g., increased intestinal tropism) that may enable the virus to invade more efficiently the intestinal target cells, leading to the enhanced enteropathogenicity of PDCoV.

## 5. Conclusions

This study isolated and serially propagated a field PDCoV strain responsible for the recent severe outbreak in South Korea. To the best of our knowledge, this is the first report corroborating the highly pathogenic potential of the KOR PDCoV isolate as naive pigs developed severe diarrhea, death, and typical macroscopic and microscopic intestinal lesions with viral antigens confirmed by DIF staining. Moreover, under the condition of this study, the clinical outcomes of PDCoV infection appear to be analogous to those caused by virulent PEDV infection. Our genetic and phylogenetic analyses indicated that the PDCoV might have undergone viral fitness to gain the highly pathogenic trait under the domestic field situation rather than introducing the virulent Chinese or SEA clade into South Korea. Because comparative analysis of paired past/recent PDCoV strains disclosed conserved in vitro phenotypes, it is likely that a combination of multiple genetic changes (i.e., point mutations) in the S1-CTD or throughout the genome contributed to the alteration in virulence; thus, cutting-edge research using reverse genetics technology will be essential for providing fundamental insights into the specific role of genetic drift in PDCoV pathogenesis. The emergence of a novel virulent variant may act as a source for large-scale epidemics, thereby reminding us of the need for monitoring and surveilling field PDCoV isolates and developing vaccines for future nationwide epizootics. The present study improved our knowledge about the pathogenic diversity of PDCoV circulating in the field. It suggested that continuous independent evolutionary processes occur to accumulate nonlethal mutations essential for viral adaptation in its mammalian host milieu.

## Figures and Tables

**Table 1:** Nucleotide and amino acid changes of GNU-2105 during serial passages in cell culture.

Genome region (nucleotide length)	Nucleotides				Amino acids			
	Position	SI	P1	P5	Position	SI	P1	P5
5′-UTR (539)	–^a^	–	–	–	–	–	–	–
ORF1a (10,881)	3,444	C	T	T	969	L	L	L
	9,567	C	G	G	3,010	P	A	A
ORF1ab (18,803)	17,015	G	T	T	5,493	G	C	C
S (3,483)	21,115	T	C	C	598	S	P	P
	21,209	T	C	C	629	F	S	S
	21,247	C	A	A	642	Q	K	K
E (252)	–	–	–	–	–	–	–	–
M (654)	–	–	–	–	–	–	–	–
NS6 (285)	–	–	–	–	–	–	–	–
N (1,029)	–	–	–	–	–	–	–	–
NS7 (603)	–	–	–	–	–	–	–	–
NS7a (303)	–	–	–	–	–	–	–	–
3′-UTR (392)	–	–	–	–	–	–	–	–

*Note*. ^a^No nucleotide or amino acid change found.

**Table 2:** Comparison of the complete genomes of the PDCoV isolates to the South Korean prototype strain KNU14-04.

Genome region (nucleotide length)	KNU-1607	GNU-2105
	% identity (no. of nt or aa differences) to KNU14-04	
5′-UTR (593)	100 (0)	99.8 (1)
ORF1ab (18,803)	99.9 (6)	99.7 (18)
nsp2 (1,779)	99.8 (1)	99.7 (2)
nsp3 (4,209)	99.9 (2)	99.5 (7)
nsp4 (1,536)	99.8 (1)	99.6 (2)
nsp5 (921)	100 (0)	99.7 (1)
nsp6 (831)	100 (0)	99.6 (1)
nsp7 (288)	99.0 (1)	100 (0)
nsp8 (567)	100 (0)	100 (0)
nsp9 (327)	99.0 (1)	100 (0)
nsp10 (399)	100 (0)	100 (0)
nsp11 (21)	86.7 (1)	100 (0)
nsp12 (2,787)	100 (0)	99.9 (1)
nsp13 (1,788)	100 (0)	99.8 (1)
nsp14 (1,551)	100 (0)	99.8 (1)
nsp15 (981)	100 (0)	99.4 (2)
nsp16 (837)	100 (0)	100 (0)
S (3,483)	99.3 (8)	99.2 (9)
E (252)	100 (0)	100 (0)
M (654)	100 (0)	100 (0)
NS6 (285)	100 (0)	98.9 (1)
N (1,029)	100 (0)	99.7 (1)
NS7 (603)	98.5 (3)	97.5 (5)
NS7a (303)	99.0 (1)	97.0 (3)
3´-UTR (392)	99.5 (2)	98.0 (8)
Total	99.8 (0/20/0)^a^	99.4 (1/35/8)^a^

*Note*. ^a^The number of individual differences in the 5′-UTR, protein-coding region, and 3′-UTR, respectively.

**Table 3:** Pairwise comparisons of the full-length genomes and the S protein genes of the GNU-2105 and global PDCoV strains.

Strain	Nucleotide/amino acid identity (%) (No. of nucleotide/amino acid differences)										
	GNU-2105	Minnesota 454	Iowa 136	HKD	Peru isolate	HKU15-155	HB	GX 1468B	NT1 1215	VN 0116	BTL 0115
GNU-2105/ KOR/2021		99.5 (131)	99.3 (165)	99.0 (248)	99.4 (153)	98.9 (288)	99.0 (257)	97.3 (688)	96.9 (782)	97.9 (546)	97.2 (705)
Minnesota 454/ USA/2014	99.2 (9)		99.8 (49)	99.4 (141)	99.5 (123)	99.2 (202)	99.3 (172)	97.5 (630)	97.1 (724)	98.1 (481)	97.4 (650)
Iowa 136/ USA/2015	99.1 (11)	99.7 (4)		99.3 (166)	99.4 (148)	99.1 (229)	99.2 (199)	97.4 (655)	97.1 (746)	98.0 (504)	97.4 (670)
HKD/ Japan/2016	98.4 (18)	98.9 (13)	98.9 (13)		99.1 (234)	98.8 (312)	98.9 (285)	97.1 (732)	96.8 (822)	97.7 (589)	97.0 (751)
Peru isolate/ Peru/2019	98.8 (14)	99.1 (11)	98.9 (13)	98.1 (22)		98.8 (303)	99.0 (264)	97.3 (694)	96.9 (792)	97.8 (552)	97.2 (717)
HKU15-155/ China/2012	98.2 (21)	98.8 (14)	98.8 (14)	98.2 (21)	98.2 (21)		99.5 (124)	97.7 (592)	97.3 (682)	98.1 (494)	97.6 (608)
HB/ China/2015	98.6 (16)	99.2 (9)	99.3 (8)	98.4 (18)	98.4 (18)	99.2 (9)		97.7 (574)	97.3 (679)	98.1 (478)	97.6 (603)
GX 1468B/ China/2018	97.4 (30)	98.0 (23)	98.0 (23)	97.4 (30)	97.5 (29)	97.6 (28)	98.0 (23)		98.0 (514)	98.6 (361)	98.3 (431)
NT1 1215/ Thailand/2015	96.7 (38)	97.3 (31)	97.3 (31)	96.6 (40)	96.7 (38)	97.2 (32)	97.5 (29)	97.4 (30)		98.1 (473)	99.4 (164)
VN 0116/ Vietnam/2015	98.7 (15)	99.1 (10)	99.1 (10)	98.4 (19)	98.5 (17)	98.4 (18)	98.7 (15)	97.8 (25)	97.2 (33)		98.4 (395)
BTL 0115/ Laos/2016	97.2 (33)	97.8 (26)	97.8 (26)	97.0 (35)	97.3 (31)	97.5 (29)	97.9 (24)	97.7 (27)	99.1 (10)	97.4 (30)	

*Note*. The percent full-length genome identities (nucleotide level) were shown in the upper right, and the percent S gene identities (amino acid level) were presented in the lower left.

## Data Availability

The data that support the findings of this study are included within the article, and the obtained full-length genome sequences were deposited in the NCBI GenBank database.

## References

[1] Jung K., Hu H., Eyerly B., Lu Z., Chepngeno J., Saif L. J. (2015). Pathogenicity of 2 porcine deltacoronavirus strains in gnotobiotic pigs. *Emerging Infectious Diseases*.

[2] Woo P. C. Y., Lau S. K. P., Lam C. S. F. (2012). Discovery of seven novel mammalian and avian coronaviruses in the genus deltacoronavirus supports bat coronaviruses as the gene source of alphacoronavirus and betacoronavirus and avian coronaviruses as the gene source of gammacoronavirus and deltacoronavirus. *Journal of Virology*.

[3] Chen Q., Gauger P., Stafne M. (2015). Pathogenicity and pathogenesis of a United States porcine deltacoronavirus cell culture isolate in 5-day-old neonatal piglets. *Virology*.

[4] Ma Y., Zhang Y., Liang X. (2015). Origin, evolution, and virulence of porcine deltacoronaviruses in the United States. *mBio*.

[5] Vitosh-Sillman S., Loy J. D., Brodersen B. (2016). Experimental infection of conventional nursing pigs and their dams with porcine deltacoronavirus. *Journal of Veterinary Diagnostic Investigation*.

[6] Jung K., Hu H., Saif L. J. (2016). Porcine deltacoronavirus infection: etiology, cell culture for virus isolation and propagation, molecular epidemiology and pathogenesis. *Virus Research*.

[7] Zhang J., Tsai Y.-L., Lee P.-Y. A. (2016). Evaluation of two singleplex reverse transcription-insulated isothermal PCR tests and a duplex real-time RT-PCR test for the detection of porcine epidemic diarrhea virus and porcine deltacoronavirus. *Journal of Virological Methods*.

[8] Hu H., Jung K., Vlasova A. N., Saif L. J. (2016). Experimental infection of gnotobiotic pigs with the cell-culture-adapted porcine deltacoronavirus strain OH-FD22. *Archives of Virology*.

[9] Jang G., Kim S.-H., Lee Y. J. (2018). Isolation and characterization of Korean porcine deltacoronavirus strain KNU16-07. *Journal of Veterinary Science*.

[10] Schoch C. L., Ciufo S., Domrachev M. (2020). NCBI Taxonomy: a comprehensive update on curation, resources and tools. *Database*.

[11] Choi S., Lee C. (2019). Functional characterization and proteomic analysis of porcine deltacoronavirus accessory protein NS7. *Journal of Microbiology and Biotechnology*.

[12] Fang P., Fang L., Liu X. (2016). Identification and subcellular localization of porcine deltacoronavirus accessory protein NS6. *Virology*.

[13] Fang P., Fang L., Hong Y. (2017). Discovery of a novel accessory protein NS7a encoded by porcine deltacoronavirus. *Journal of General Virology*.

[14] Marthaler D., Raymond L., Jiang Y., Collins J., Rossow K., Rovira A. (2014). Rapid detection, complete genome sequencing, and phylogenetic analysis of porcine deltacoronavirus. *Emerging Infectious Diseases*.

[15] Lee S., Lee C. (2014). Complete genome characterization of Korean porcine deltacoronavirus strain KOR/KNU14-04/2014. *Genome Announcements*.

[16] Li G., Chen Q., Harmon K. M. (2014). Full-length genome sequence of porcine deltacoronavirus strain USA/IA/2014/8734. *Genome Announcements*.

[17] Wang L., Byrum B., Zhang Y. (2014). Detection and genetic characterization of deltacoronavirus in pigs, Ohio, USA, 2014. *Emerging Infectious Diseases*.

[18] Dong N., Fang L., Zeng S., Sun Q., Chen H., Xiao S. (2015). Porcine deltacoronavirus in mainland China. *Emerging Infectious Diseases*.

[19] Janetanakit T., Lumyai M., Bunpapong N. (2016). Porcine deltacoronavirus, Thailand, 2015. *Emerging Infectious Diseases*.

[20] Saeng-Chuto K., Stott C. J., Wegner M., Senasuthum R., Tantituvanont A., Nilubol D. (2017). Retrospective investigation and evolutionary analysis of a novel porcine deltacoronavirus strain detected in Thailand from 2008 to 2015. *Archives of Virology*.

[21] Suzuki T., Shibahara T., Imai N., Yamamoto T., Ohashi S. (2018). Genetic characterization and pathogenicity of Japanese porcine deltacoronavirus. *Infection, Genetics and Evolution*.

[22] Lee S., Park G.-S., Shin J.-H., Lee C. (2014). Full-genome sequence analysis of a variant strain of porcine epidemic diarrhea virus in South Korea. *Genome Announcements*.

[23] Jang G., Lee K.-K., Kim S.-H., Lee C. (2017). Prevalence, complete genome sequencing and phylogenetic analysis of porcine deltacoronavirus in South Korea, 2014–2016. *Transboundary and Emerging Diseases*.

[24] Kim S. H., Pajarillo E. A. B., Balolong M. P., Lee J. Y., Kang D.-K. (2016). Constructing proteome reference map of the porcine jejunal cell line (IPEC-J2) by label-free mass spectrometry. *Journal of Microbiology and Biotechnology*.

[25] Jeon J. H., Lee Y. J., Lee C. (2020). Porcine deltacoronavirus activates the Raf/MEK/ERK pathway to promote its replication. *Virus Research*.

[26] Jeon J. H., Lee C. (2021). Stress-activated protein kinases are involved in the replication of porcine deltacoronavirus. *Virology*.

[27] Lee S., Kim Y., Lee C. (2015). Isolation and characterization of a Korean porcine epidemic diarrhea virus strain KNU-141112. *Virus Research*.

[28] Lee S., Lee C. (2018). Genomic and antigenic characterization of porcine epidemic diarrhoea virus strains isolated from South Korea, 2017. *Transboundary and Emerging Diseases*.

[29] Lee S., Lee D.-U., Noh Y.-H. (2019). Molecular characteristics and pathogenic assessment of porcine epidemic diarrhoea virus isolates from the 2018 endemic outbreaks on Jeju Island, South Korea. *Transboundary and Emerging Diseases*.

[30] Jang G., Park J., Lee C. (2021). Successful eradication of porcine epidemic diarrhea in an enzootically infected farm: a two-year follow-up study. *Pathogens*.

[31] Park J., Lee C. (2020). Emergence and evolution of novel G2b-like porcine epidemic diarrhea virus inter-subgroup G1b recombinants. *Archives of Virology*.

[32] Sagong M., Lee C. (2011). Porcine reproductive and respiratory syndrome virus nucleocapsid protein modulates interferon-*β* production by inhibiting IRF3 activation in immortalized porcine alveolar macrophages. *Archives of Virology*.

[33] Reed L. J., Muench H. (1938). A simple method of estimating fifty percent endpoints. *American Journal of Epidemiology*.

[34] Hou Y., Lin C.-M., Yokoyama M. (2017). Deletion of a 197-amino-acid region in the N-terminal domain of spike protein attenuates porcine epidemic diarrhea virus in piglets. *Journal of Virology*.

[35] Jang G., Min K.-C., Lee I. H. (2023). Deletion of pentad residues in the N-terminal domain of spike protein attenuates porcine epidemic diarrhea virus in piglets. *Veterinary Microbiology*.

[36] Li W., van Kuppeveld F. J. M., He Q., Rottier P. J. M., Bosch B.-J. (2016). Cellular entry of the porcine epidemic diarrhea virus. *Virus Research*.

[37] Lee Y. N., Lee C. (2013). Complete genome sequence of a novel porcine parainfluenza virus 5 isolate in Korea. *Archives of Virology*.

[38] Thompson J. D., Gibson T. J., Plewniak F., Jeanmougin F., Higgins D. G. (1997). The CLUSTAL_X windows interface: flexible strategies for multiple sequence alignment aided by quality analysis tools. *Nucleic Acids Research*.

[39] Saitou N., Nei M. (1987). The neighbor-joining method: a new method for reconstructing phylogenetic trees. *Molecular Biology and Evolution*.

[40] Kumar S., Stecher G., Li M., Knyaz C., Tamura K. (2018). MEGA X: Molecular evolutionary genetics analysis across computing platforms. *Molecular Biology and Evolution*.

[41] Lee S., Son K.-Y., Noh Y.-H. (2017). Genetic characteristics, pathogenicity, and immunogenicity associated with cell adaptation of a virulent genotype 2b porcine epidemic diarrhea virus. *Veterinary Microbiology*.

[42] Jang G., Won H., Lee D.-U. (2019). Assessment of the safety and efficacy of an attenuated live vaccine based on highly virulent genotype 2b porcine epidemic diarrhea virus in nursing piglets. *Veterinary Microbiology*.

[43] Jung K., Kim J., Ha Y., Choi C., Chae C. (2006). The effects of transplacental porcine circovirus type 2 infection on porcine epidemic diarrhoea virus-induced enteritis in preweaning piglets. *The Veterinary Journal*.

[44] Yuan Y., Zu S., Zhang Y., Zhao F., Jin X., Hu H. (2021). Porcine deltacoronavirus utilizes sialic acid as an attachment receptor and trypsin can influence the binding activity. *Viruses*.

[45] Zhang J., Chen J., Shi D. (2019). Porcine deltacoronavirus enters cells via two pathways: A protease-mediated one at the cell surface and another facilitated by cathepsins in the endosome. *Journal of Biological Chemistry*.

[46] Zhang H., Han F., Shu X. (2022). Co-infection of porcine epidemic diarrhoea virus and porcine deltacoronavirus enhances the disease severity in piglets. *Transboundary and Emerging Diseases*.

[47] Jung K., Hu H., Saif L. J. (2017). Calves are susceptible to infection with the newly emerged porcine deltacoronavirus, but not with the swine enteric alphacoronavirus, porcine epidemic diarrhea virus. *Archives of Virology*.

[48] Liang Q., Zhang H., Li B. (2019). Susceptibility of chickens to porcine deltacoronavirus infection. *Viruses*.

[49] Boley P. A., Alhamo M. A., Lossie G. (2020). Porcine deltacoronavirus infection and transmission in poultry, United States. *Emerging Infectious Diseases*.

[50] Zhang H., Ding Q., Yuan J., Han F., Wei Z., Hu H. (2022). Susceptibility to mice and potential evolutionary characteristics of porcine deltacoronavirus. *Journal of Medical Virology*.

[51] Lednicky J. A., Tagliamonte M. S., White S. K. (2021). Independent infections of porcine deltacoronavirus among Haitian children. *Nature*.

[52] Anon (2014). USDA to require reports of PED. *Journal of the American Veterinary Medical Association*.

[53] Saeng-Chuto K., Jermsutjarit P., Stott C. J., Vui D. T., Tantituvanont A., Nilubol D. (2020). Retrospective study, full-length genome characterization and evaluation of viral infectivity and pathogenicity of chimeric porcine deltacoronavirus detected in Vietnam. *Transboundary and Emerging Diseases*.

[54] Song D., Zhou X., Peng Q. (2015). Newly emerged porcine deltacoronavirus associated with diarrhoea in swine in China: identification, prevalence and full-length genome sequence analysis. *Transboundary and Emerging Diseases*.

[55] Li W., Hulswit R. J. G., Kenney S. P. (2018). Broad receptor engagement of an emerging global coronavirus may potentiate its diverse cross-species transmissibility. *Proceedings of the National Academy of Sciences of the United States of America*.

[56] Ji W., Peng Q., Fang X. (2022). Structures of a deltacoronavirus spike protein bound to porcine and human receptors. *Nature Communications*.

[57] Shang J., Zheng Y., Yang Y. (2018). Cryo-electron microscopy structure of porcine deltacoronavirus spike protein in the prefusion state. *Journal of Virology*.

[58] Liu Y., Wang B., Liang Q.-Z. (2021). Roles of two major domains of the porcine deltacoronavirus S1 subunit in receptor binding and neutralization. *Journal of Virology*.

[59] Wang B., Liu Y., Ji C.-M. (2018). Porcine deltacoronavirus engages the transmissible gastroenteritis virus functional receptor porcine aminopeptidase N for infectious cellular entry. *Journal of Virology*.

